# Concurrent administration of IFNα14 and cART in TKO-BLT mice enhances suppression of HIV-1 viremia but does not eliminate the latent reservoir

**DOI:** 10.1038/s41598-019-54650-9

**Published:** 2019-12-02

**Authors:** Kathrin Sutter, Kerry J. Lavender, Ronald J. Messer, Marek Widera, Katie Williams, Brent Race, Kim J. Hasenkrug, Ulf Dittmer

**Affiliations:** 1 Institute for Virology, University Hospital Essen, University of Duisburg-Essen, 45122 Essen, Germany; 2 Laboratory of Persistent Viral Diseases, Rocky Mountain Laboratories, NIAID, NIH, Hamilton, MT USA; 3 Present Address: Department of Biochemistry, Microbiology & Immunology, College of Medicine, University of Saskatchewan, Saskatoon, SK Canada

**Keywords:** Viral reservoirs, Restriction factors

## Abstract

Combination antiretroviral therapy (cART) prevents HIV-1 replication but does not eliminate the latent reservoir and cure the infection. Type I interferons (IFN) mediate antiviral effects through different mechanisms than cART. We previously showed that IFNα14 is the most potent IFNα subtype against HIV-1 and that it can significantly reduce the HIV-1 proviral reservoir. This study sought to determine whether combining cART with IFNα14 therapy would produce greater reductions in HIV-1 viral and proviral loads than ART alone. Immunodeficient *Rag2*^−/−^*γ*_*c*_^−/−^*CD47*^−/−^ C57BL/6 mice were humanized by the BLT method, infected with HIV-1_JR-CSF_ and the *in vivo* efficacy of cART was compared with combined cART/IFNα14 therapy. Infection was allowed to establish for 6 weeks prior to 4 weeks of treatment with oral cART either with or without IFNα14. Plasma viral RNA and splenic CD4^+^ T cell viral DNA levels were measured immediately after treatment and after 2 weeks of therapy interruption. Augmentation of cART with IFNα14 resulted in significantly enhanced suppression of HIV-1 plasma viremia. However, no significant reduction in total viral DNA was detectable. Furthermore, virus rebounded after treatment interruption to similar levels in both groups. Thus, augmentation of cART with IFNα14 resulted in a more pronounced reduction of HIV viremia levels over cART alone, but the effect was not potent enough to be detected at the viral DNA level or to prevent virus rebound following therapy interruption in immune system-humanized mice.

## Introduction

The major obstacle in developing a sterilizing or functional cure for HIV-1 infection is the presence of a reservoir of latently infected cells. Although combination antiretroviral therapy (cART) can efficiently interfere with active virus replication and suppress viremia to very low or undetectable levels, it cannot purge the reservoir of latently infected cells. Thus, cART interruption ultimately leads to reactivation of the reservoir and viremia. Many cure strategies aim to harness aspects of the host immune response to clear latently infected cells or to induce immunological control upon treatment interruption. The testing and development of such strategies are facilitated by the use of small animal models, such as the triple knockout bone marrow-liver-thymus (TKO-BLT)-humanized mouse model^1,2^. TKO-BLT mice become reconstituted with high levels of multi-lineage human hematopoietic cells, are susceptible to HIV-1 infection, and develop hallmarks of human HIV-1 infection such as hyper-immune activation and CD4^+^ T cell depletion. They are also resistant to graft versus host disease so they can be studied relatively long-term in the absence of an underlying immunopathological condition^1,2^. Like other BLT models, TKO-BLT mice can be infected with HIV-1 and treated with ART to establish latent HIV-1 infection, which rapidly recrudesces after treatment interruption^3,4^. Importantly for cure studies that rely on endogenous immunity, BLT mice also have functional immune responses including HIV-1-specific responses^1,5,6^.

We previously showed that a specific interferon alpha (IFNα) subtype, IFNα14, mediated superior suppression of HIV-1 infection compared to other subtypes in TKO-BLT mice^7^. Interestingly, both intrinsic and innate immunity were associated with the anti-HIV-1 activity mediated by IFNα14. For example, increased levels of signature APOBEC3G mutations were found in proviral DNA, potentially reducing the replicative fitness of the reservoir. Furthermore, IFNα14 specifically activated NK cells, which may have eliminated virally infected cells. It was therefore an attractive strategy to administer IFNα14 in conjunction with cART to harness their combined and divergent mechanisms of action in order to potentially produce reductions in the size and/or fitness of the latent reservoir. The use of IFNα in the treatment of HIV-1 or as a component of a cure strategy is controversial, particularly due to recent studies blocking the IFNα/β receptor (IFNAR) in HIV-1 infected humanized mice. One study using IFNAR blockade demonstrated that, despite having antiviral effects, IFNAR signaling may drive CD4^+^ T cell depletion and dysfunction of CD4^+^ and CD8^+^ T cells during chronic infection^8^. Additionally, Zhen and colleagues reported that ART combined with IFNAR blockade in HIV-1 infected BLT mice decreased plasma RNA levels as well as numbers of latently infected cells^9^. In contrast to these studies that block all type I IFN-mediated effects, we and others have reported that specific IFNα subtypes can mediate beneficial effects in HIV-1 infected humanized mice^7,10^. This discrepancy in methodology is worth consideration as seminal work in the chronic LCMV mouse model has shown that despite signaling through the same receptor, only IFNβ and not IFNα impaired antiviral immunity and supported persistent infection^11^. Thus, subtype specific IFNα treatment of HIV-1 infection remains a viable therapeutic option and is worth pursuing, as evidenced by continued HIV-1 clinical trials aiming to harness the potent effects of IFNα on the HIV-1 reservoir. Such studies in HIV-1 infected subjects are currently underway using the IFNα2 subtype (NCT02227277). Unfortunately, the use of the IFNα2 subtype is based on its current approval status for clinical use against hepatitis viruses, but it has mostly been shown to have low antiviral activity against HIV-1 *in vitro*^12^ and *in vivo*^7,13–17^. In HIV-1 patients co-infected with HCV, short-term treatment with cART and IFNα2 reduced HIV-1 expression and decreased CD4+ T cell activation^18^. In all the studies published so far, the most potent human IFNα subtype against HIV-1 has been IFNα14^7,10,12^. We therefore used the TKO-BLT human immune system mouse model to assess whether the addition of IFNα14 therapy to cART during suppression of viral replication would produce a smaller and/or less replication competent latent HIV reservoir.

## Materials and Methods

### Humanized TKO-BLT mice

Male and female C57BL/6 Rag2−/−γc−/−CD47−/− (TKO) mice were humanized using the bone marrow, liver, thymus (BLT) method as previously described [2]. Animals were housed under specific pathogen-free conditions. All animal studies were performed under an AAALAC-accredited Rocky Mountain Laboratories, National Institute of Allergy and Infectious Diseases, National Institutes of Health (USA) Institutional Animal Care and Use Committee-approved animal study protocol in accordance with the regulations and guidelines of the Animal Care and Use Committee of the Rocky Mountain Laboratories, NIAID, NIH. The study included provisions to ensure proper anesthesia during procedures, pain relief medication during recovery and daily health monitoring. The use of donated anonymous fetal tissue biospecimens for the research described in this paper was approved by the National Institutes of Health (NIH) Office of Human Subjects Research Protection (OHSRP Project ID# P194542; Ref. 526739). Donor tissues for humanization were obtained with informed consent following all guidelines and regulations of NIH and the Office of Human Subjects Research Protection. All research involving human fetal tissue was completed before June 5, 2019.

### HIV-1 challenge, cART and cART plus IFNα14 treatment

R5-tropic HIV-1_JR-CSF_ stocks were prepared and inoculated intraperitoneally as previously described^1^. TZM-bl (JC53-bl) reporter cells (NIH AIDS Research and Reference Reagent Program from Drs John Kappes and Xiaoyun Wu and Tranzyme, Inc.) were used to determine stock concentrations. Infected mice were assigned to groups with similar mean p24 antigenemia at 6 weeks post infection (wpi). Starting at 6 weeks post infection, mice were free-fed with cART-supplemented tenofovir (TDF; Gilead Sciences, Foster City, CA, USA), emtricitabin (FTC; Gilead Sciences) and raltegravir (RAL; Merck & Co., Kenilworth, NJ, USA) mouse chow as previously described^4^. One group of mice additionally received intraperitoneal injections of 1.5 × 10^5^U/mouse IFNα14 after 6 weeks of infection and subsequently at 24 h intervals for 4 weeks.

### Quantification of HIV-1 RNA and DNA

Plasma HIV RNA was isolated with the QIAamp Viral RNA Kit (Qiagen, Hilden, Germany) and quantified using the Abbott RealTime HIV-1 m2000 test system as described by the manufacturers. By using an input volume of 100 µl plasma the detection limit was 300 copies/ml. Total HIV-1 DNA was quantified performing a pre-PCR followed by a probe-based real-time PCR approach as described previously^19,20^. Briefly, genomic DNA was isolated from splenic CD4^+^ cells using QIAamp DNA Mini Kit (Qiagen). CD3 and HIV-1 DNA sequences were pre-amplified by carrying out 12 cycling steps in a TProfessional TRIO Thermocycler (Biometra, Goettingen, Germany). The pre-PCR amplicons were diluted and subjected to quantitative real-time PCR analysis using the Rotor-Gene Probe PCR Kit (Qiagen) performed in a Rotor-Gene Q instrument (Qiagen). TaqMan probes for CD3 and HIV-1-DNA quantification were dually labelled with YAK-BHQ-1 and 6-FAM-BHQ-1, respectively. Standard curves were generated with plasmid DNA templates harboring the corresponding amplicon regions or genomic DNA of HIV-1 LTR-harbouring cells for the quantification of integrated provirus^20^. Determinations of HIV infection levels in plasma for group assignments were done by p24 ELISA (Advanced Bioscience Laboratories, Rockville MD).

### Recombinant IFNα14

Production of human IFNα14 protein was previously described^7^ and endotoxin levels were less than 0.0025 endotoxin units (EU)/ml (ToxinSensor; Genscript, Piscataway, NJ). The biological activity of the recombinantly produced IFNα14 was measured with an IFN-stimulated response element (ISRE) based  reporter assay and normalized to commercially available IFNα14 (PBL Assay Science, Piscataway Township, NJ, USA), as described elsewhere^7^.

## Results and Discussion

**Figure 1:**
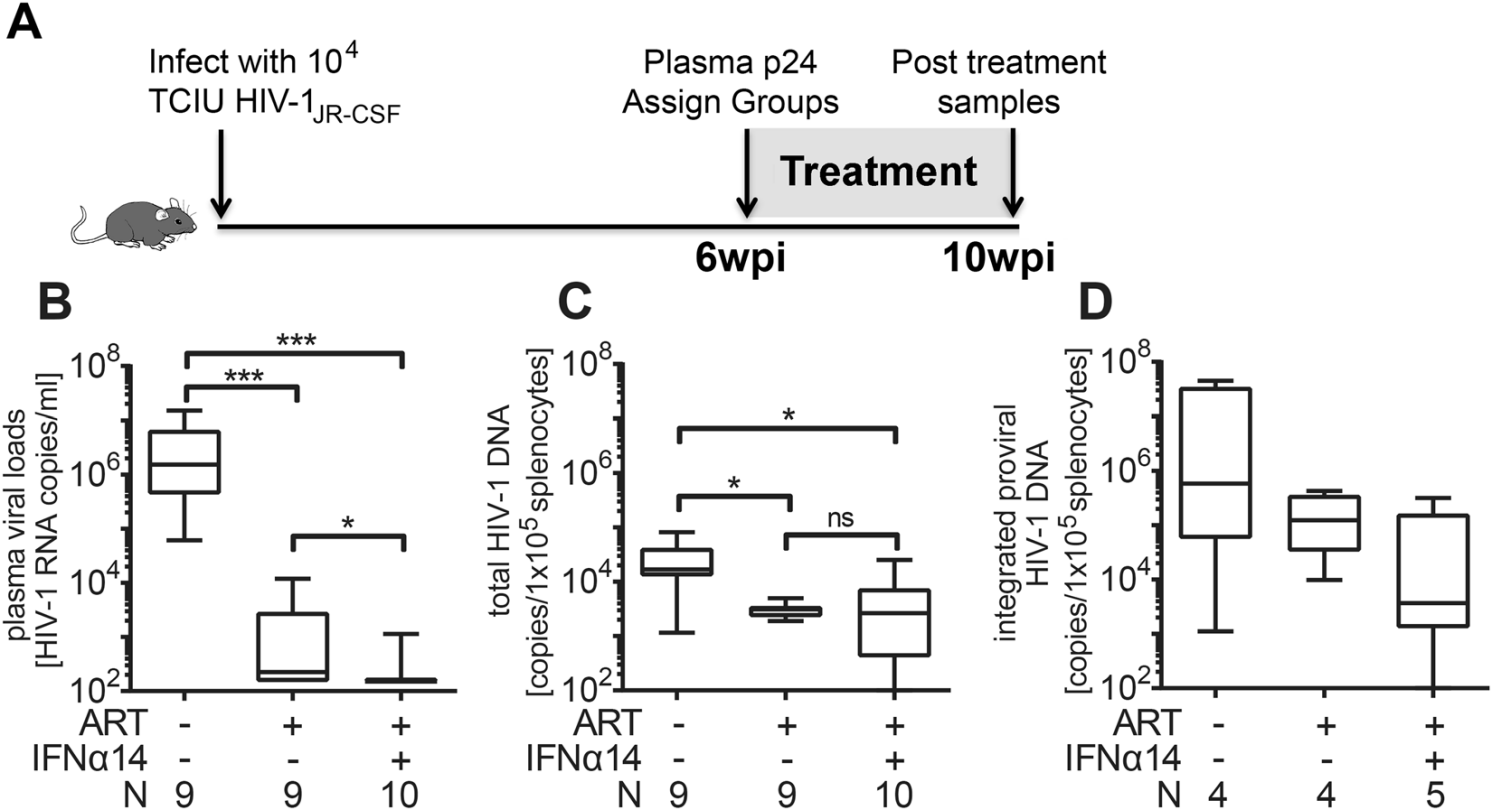
Addition of IFNα14 to cART therapy for 4 weeks significantly reduces plasma viral RNA but does not decrease cellular viral DNA levels. (**A**) Scheme of the experimental timeline. Mice were inoculated i.p. with 1 × 10^4^ TCIUs of HIV-1_JR-CSF_ and infection was allowed to progress for 6 weeks. At 6wpi p24-CA levels were determined and mice were assigned to groups with comparable HIV-1 antigen loads. Mice were either free-fed with cART chow (n = 18), given cART chow plus daily i.p. injections of 1.5 × 10^5^ units of IFNα14 (n = 20) or left untreated (n = 18) for 4 weeks (10wpi). (**B**) Levels of HIV-1 viral RNA in plasma were measured directly after analytical treatment interruption (10wpi). Student’s t test; * < 0.05, ns = not significant. (**C**) Levels of total HIV-1 viral DNA and (**D**) levels of integrated proviral HIV-1 DNA from CD4^+^ enriched splenocytes were determined (10wpi). One-way ANOVA with Tukey’s post-test (untreated n = 9, ART n = 9, ART + IFNα14 n = 10). Box and whisker plots depict means with standard deviations and ranges. N = numbers of mice investigated.

Infection of humanized TKO-BLT mice^1,2^ was done by intraperitoneal inoculation of 10^4^ tissue culture infectious units (TCIU) of HIV-1_JR-CSF_. The HIV-1 infection was allowed to progress for 6 weeks prior to initiation of treatment with cART or cART plus IFNα14 (Fig. 1A). This schedule was chosen to allow HIV-1 infection to become well established and to represent a reasonable time frame relative to human patients seeking clinical treatment for symptomatic HIV-1 infections. Since the dosage of IFNα that can be used clinically is limited by undesirable side effects^21^, we used the mouse equivalent^22^ units of IFNα14 used to treat melanoma patients with IFNα2^23^, which was previously shown to suppress HIV-1 replication and reduce proviral loads in TKO-BLT mice^7^.

At 6 weeks post infection all mice were tested for plasma p24 levels and placed in three groups with closely equivalent mean HIV-1 p24 antigen levels. Virus suppression was initiated in two of the groups using free feeding of drugs incorporated into mouse chow. One of the cART groups was also treated by daily injections of IFNα14 for 4 weeks. One day after the cessation of the 4 week treatment period, all mice were analyzed for plasma viral RNA (Fig. 1B). The experiment was repeated in two independent cohorts of mice that gave very similar results so the data were combined for analysis. Results showed that the addition of IFNα14 therapy to cART resulted in a statistically significant 8-fold reduction in plasma virus RNA levels compared to cART alone (Fig. 1B).

In HIV-1 infected humans, cART alone cannot purge the latent DNA reservoir but has been reported to reduce cellular HIV-1 DNA levels over the first year of therapy^24^. To analyze DNA levels in the mice following cART, a subset of mice from each group was euthanized to harvest spleens for isolation of CD4^+^ T cell DNA. Both of the treatment arms produced a statistically significant reduction in the mean HIV-1 DNA levels compared to the untreated control group, but the addition of IFNα14 therapy to cART did not result in any further reduction in total splenic viral DNA (Fig. 1C). The viral reservoir size is better described by measuring integrated HIV provirus levels than total HIV DNA. Unfortunately, we could perform such assays for only 4–5 mice per group due to sample size limitations. ART treatment alone (median: 1.2 × 10^5^) did not significantly change copy numbers of integrated HIV DNA compared to HIV-infected, untreated controls (5.8 × 10^5^) (Fig. 1D). In contrast, the combination therapy of ART and IFNα14 reduced proviral DNA levels by more than two logs (3.6 × 10^3^). However, due to the small sample size this difference was not statistically significant. The data nevertheless suggested that the combination therapy might affect the viral reservoir size of HIV.

**Figure 2:**
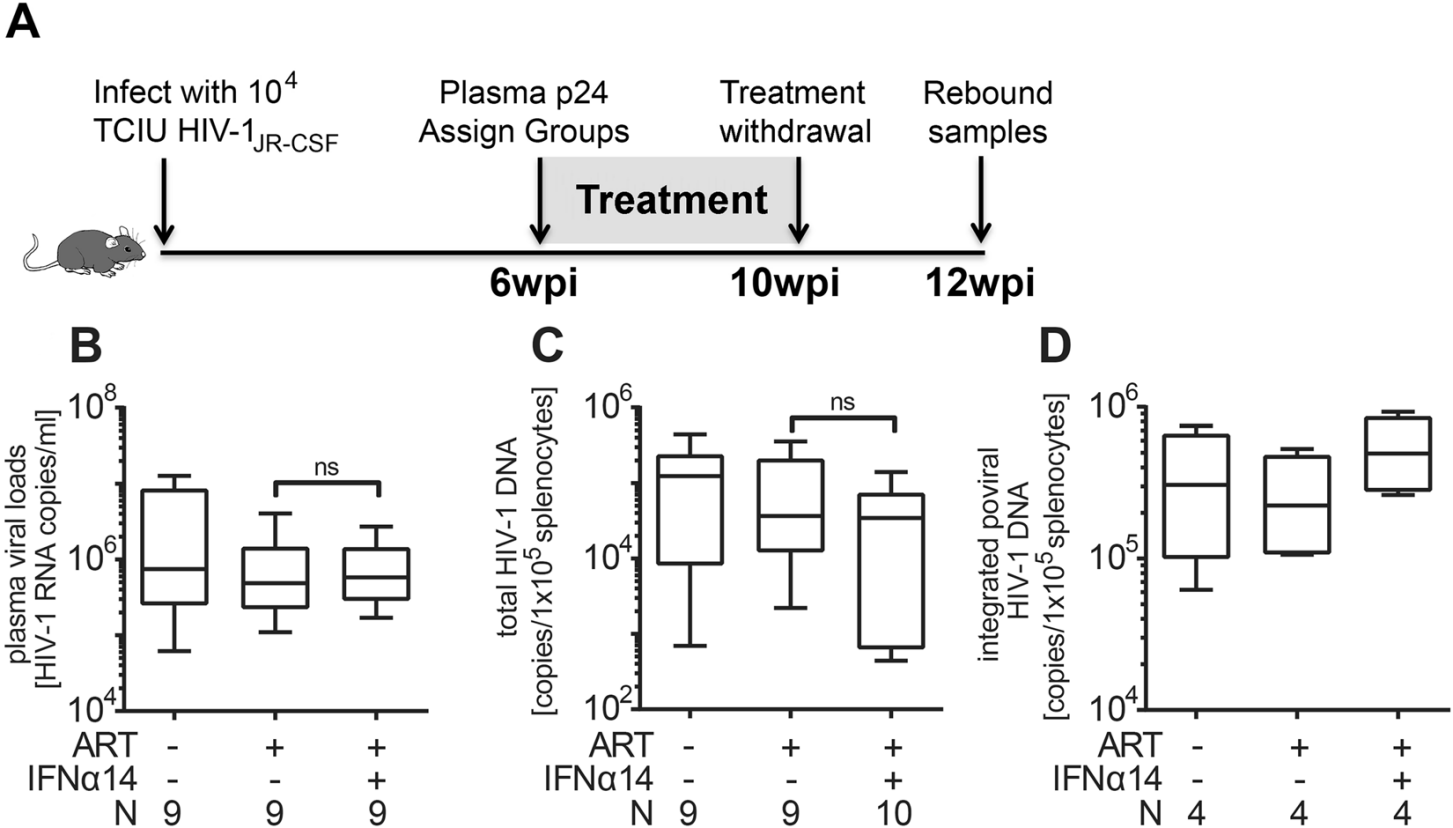
HIV-1 viremia and viral recrudesce in cART or cART plus IFNα14 treated mice after 2 weeks of treatment withdrawal. (**A**) Schematic drawing of the experimental timeline. Mice were inoculated i.p with 1 × 10^4^ TCIUs of HIV-1_JR-CSF_ and infection was allowed to progress for 6 weeks. At 6wpi mice were either free-fed with cART chow, given cART chow plus daily i.p. injections of IFNα14 or left untreated for 4 weeks. At 10wpi all treatment was discontinued (analytical treatment interruption) for additional 2 weeks prior to sample collection at 12wpi. (**B**) Levels of HIV-1 viral RNA in plasma (untreated n = 9, ART n = 9, ART + IFNα14 n = 9) and (**C**) levels of total HIV-1 viral DNA and (**D**) levels of integrated proviral HIV-1 DNA in CD4^+^ enriched splenocytes (untreated n = 9, ART n = 9, ART + IFNα14 n = 10) were analysed. Box and whisker plots depict means with standard deviations and ranges. One-way ANOVA with Tukey’s post-test; ns = not significant. N = numbers of mice investigated.

Because many proviruses may be defective, the HIV-1 DNA content does not reflect the number of transcriptionally active and/or virus-producing cells. Additionally, we and others^7,12^ have previously shown that IFNα14 induces increased levels of APOBEC3G signature mutations in HIV-1 that could affect viral fitness without altering total DNA levels. Thus, we used analytical cART interruption to evaluate virus rebound, looking for potential IFNα14-associated reductions or delays in viral recrudescence. cART was discontinued for 2 weeks after treatment (Fig. 2A) and the animals were analyzed for viral RNA levels (Fig. 2B). No significant differences between the cART-only and the cART plus IFNα14 groups were observed. At 2 weeks post-cART interruption, the HIV-1 viral RNA levels (Fig. 2B) as well as total DNA (Fig. 2C) and integrated proviral DNA levels (Fig. 2D) were almost as high as in the untreated controls and not significantly different from each other. Thus, addition of IFNα14 therapy to cART during established HIV-1 infection provided no observable long-lasting benefit in preventing virus rebound following therapy interruption.

The current results reveal that cART plus IFNα14 therapy may be more efficacious than cART alone in reducing HIV-1 plasma viral loads (Fig. 1B). Thus, it could be used for patients who are refractory to full virus suppression from cART alone, patients who develop resistance mutations affecting multiple ART drug classes, or during periods of cART interruption. However, the concurrent administration of IFNα14 and cART during the induction of HIV-1 latency in humanized TKO-BLT mice at the doses and treatment duration tested, had no effect on the replication competent HIV-1 reservoir as evidenced by no reduction in the amount of cellular viral DNA, and no reduction or delay in viral recrudescence upon treatment interruption. It is quite possible that cART suppression of virus replication might have limited the antiviral effect of the IFNα14 *in vivo* if active viral replication is required for IFNα14 to mediate its effect on the DNA reservoir. This could occur, for example, if IFNα14-activated NK cells could not recognize and kill latently infected cells. Since these experiments were performed in humanized mice rather than humans, there may have been anomalous interactions between IFNα14 and mouse cells resulting in a failure of the combination therapy in effecting the viral reservoir. For example, IFNα14 has been shown to activate NK cells in HIV infections, but the levels of NK cells in TKO-BLT-humanized mice are not as high as they are in humans and may not be able to exert a sufficient antiviral effect^7^. It is also possible that testing of additional mice might produce statistically significant effects especially with longer treatment schedules or in combination with latency reversal drugs. Although these negative results are not encouraging for HIV cure, IFNα subtypes remain interesting drug candidates for HIV-1 therapy because of their multifunctional antiviral properties, including the possible re-activation of latent HIV, the induction of HIV restriction factors, protection of uninfected host cells against HIV infection (antiviral state), and immune-stimulatory activities.
